# Immobilization of poly(vinyl pyrrolidone) in Polysulfone Membranes by Radically-Initiated Crosslinking Using Potassium Persulfate

**DOI:** 10.3390/membranes12070664

**Published:** 2022-06-28

**Authors:** Danae Gonzalez Ortiz, Morgan Nouxet, William Maréchal, Olivier Lorain, André Deratani, Céline Pochat-Bohatier

**Affiliations:** 1 Institut Européen des Membranes, IEM UMR 5635, Université de Montpellier, CNRS, ENSCM, 34095 Montpellier, France; danae.gonzales-ortiz@umontpellier.fr (D.G.O.); morgan.nouxet@umontpellier.fr (M.N.); andre.deratani@umontpellier.fr (A.D.); 2 Polymem, 3 Rue de l’Industrie, 31320 Castanet-Tolosan, France; w.marechal@polymem.fr (W.M.); o.lorain@polymem.fr (O.L.)

**Keywords:** polymer membranes, polysulfone, hydrophilic additive, polyvinylpyrrolidone, radical initiated crosslinking

## Abstract

Polysulfone (PSU) membranes with poly(vinyl pyrrolidone) (PVP) as a pore-forming and hydrophilic additive were prepared using the non-solvent-induced phase separation (NIPS) technique. PVP immobilization by radical-initiated crosslinking using potassium persulfate (KPS) was studied in view of obtaining membranes with high and long-lasting surface hydrophilicity. A method based on the ATR-FTIR technique was developed to discriminate crosslinked PVP from unreacted PVP in the membrane. The crosslinking progress was investigated as a function of temperature, KPS concentration, and reaction time. The results showed that temperature was the main factor influencing the crosslinking reaction since radical formation is temperature-dependent. Increasing the concentration of KPS and the reaction time led to an increase in the crosslinking rate. The effect of the degree of PVP crosslinking on the structure and properties of the prepared membranes was examined by studying mechanical properties, morphology by SEM, surface hydrophilicity by contact angle measurements, and water permeability.

## 1. Introduction

Presently, membrane technologies are important separation techniques, especially for water treatment [1]. Among all commercially available polymers, polysulfone (PSU) is one of the most commonly used for ultrafiltration (UF) membranes. This polymer stood out for its excellent chemical and thermal resistance during membrane washing and its good mechanical properties suitable for long-term use. The main drawback of PSU membranes is their inherent hydrophobic behavior, which often results in serious fouling when in contact with biomolecules present in natural waters and/or hydrophobic molecules in wastewaters. Membrane manufacturers overcome this problem by incorporating hydrophilic additives in the dope solution before membrane preparation by phase separation. This reduces the hydrophobicity of the material and thus allows the preparation of membranes with improved fouling resistance. Surface modification techniques on preformed membranes such as coating, blending, or grafting have been also reported [2].

Poly(vinyl pyrrolidone) (PVP) is a low molecular weight polymer frequently added in the collodion formulation to induce pore formation and increase membrane hydrophilicity [3,4]. PVP is a water-soluble polymer, non-toxic, chemically inert, pH-stable, and biocompatible. Due to its inherent hydrophilicity, PVP tends to solubilize in water and a large amount of this polymer can leach out from the membrane during membrane preparation, rising the membrane’s porosity. However, a part of the PVP is expected to remain in the bulk of the manufactured membrane to increase its hydrophilicity. Without covalent binding, the PVP only bound by weak interactions tends to be released during membrane operation, first resulting in a deterioration of membrane hydrophilicity, but also possibly blocking the pores and reducing the initial water flux [5,6]. The immobilization of PVP, especially on the membrane surface, is a way to prevent the PVP from leaching out of the membranes and to maintain hydrophilicity on the membrane active layer. For this purpose, an additional step consisting of the crosslinking of the PVP is needed to ensure the immobilization of the polymer in the membranes. Indeed, PVP can be crosslinked using different treatments such as γ-ray [7,8,9] or UV irradiation [10,11,12,13], heat treatment [14,15], or using chemical agents, i.e., inorganic persulfate [16,17]. Among these techniques, persulfate-initiated crosslinking offers the advantages of being easy to implement in existing facilities and providing non-hazardous decomposition products.

To the best of our knowledge, no studies have yet reported crosslinking of PVP using persulfate on PSU membranes. However, reference can be made to studies conducted on surface-modified polyvinylidene fluoride (PVDF) membranes. Bi et al. prepared PVDF/PVP hollow fibers microporous membranes by immobilizing the PVP via chemical crosslinking using potassium persulfate as an initiator agent. The authors studied the effect of PVP load (0.1–10 wt.%) and crosslinking reaction time (2–24 h) at 90 °C on the performance of the membranes. The results showed an increase in the hydrophilicity, and the water contact angle measured 45 s after drop deposition is close to zero. The pure water flux of PVDF/PVP membranes with 5% PVP load and 6 h reaction time was 600 L m^−2^ h^−1^. The membranes exhibited good stability since just a small amount of PVP leached out of the membrane [18]. In another study, flat-sheet PVDF/PVP membranes were prepared through a two-step surface grafting method in order to enhance the antifouling properties. First, the PVDF raw commercial membrane was immersed in a 7 wt.% PVP solution at 60 °C for 12 h. Then, the PVP-modified PVDF membrane was soaked in a solution containing 5 wt.% PVP and 0.4 wt.% KPS at 90 °C for 6 h. This method, consisting of grafting of a PVP coating allowed for the modification of both the surface and cross-section of the membranes, resulting in pore and surface modifications. Due to pore modification, the membrane flux was enhanced up to 171 L m^−2^ h^−1^, while surface modification increased the hydrophilicity of the membrane [19]. However, it can be noticed that in both studies, the cross-linking reaction was conducted at a quite high temperature (90 °C), and consequently induced high energy consumption.

In this study, PSU flat-sheet microporous membranes were prepared using PVP as a pore-forming agent but also as an additive to increase the hydrophilicity. The immobilization of the residual PVP on the outer surface and in the cross-section of PSU membranes was performed via a cross-linking reaction using potassium persulfate (K_2_S_2_O_8_) directly after phase separation without a subsequent coating. Different reaction conditions were investigated in order to find the optimum conditions to improve the surface membrane hydrophilicity with consideration to the issue of energy consumption: temperature, reaction time, and crosslinking agent concentration. The monitoring of PVP leaching and PVP immobilization was analyzed by a method based on attenuated total reflectance Fourier transform infrared (ATR–FTIR) spectroscopy. The hydrophilicity of the surface membrane was investigated by contact angle (CA) measurements and the morphology of the PSU/PVP membranes was observed by scanning electron microscopy (SEM). The water permeability and the mechanical properties of the modified membranes were reported to illustrate the membrane performance.

## 2. Experimental Part

### 2.1. Materials

**Figure 1:**
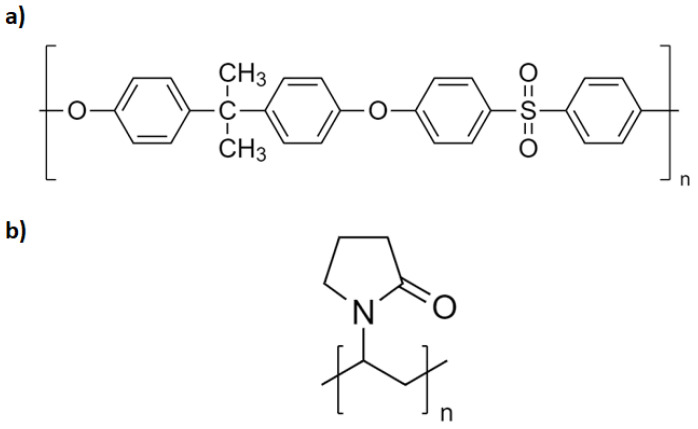
Chemical structures of (**a**) polysulfone and (**b**) polyvinylpyrrolidone.

Polysulfone (PSU) (CAS n°25135-51-7) MW 75K (Figure 1a), and polyvinylpyrrolidone (PVP, CAS n° 9003-39-8) MW 30K (Figure 1b) were supplied by Solvay, Lyon, France. N-methyl pyrrolidone 99.5%, (NMP, CAS n°872-50-4) and potassium persulfate (K_2_SO_4_, CAS n° 7727-21-1) ACS reagent ≥ 99.0% were provided by Sigma Aldrich, Saint-Quentin-Fallavier, France. In all experiments, the materials were used as received. Ultra-pure water (18.2 mΩ) was used to carry out the experiments.

### 2.2. Methods

#### 2.2.1. Preparation of Polymer Dope Solution

Polymer dope solution was prepared in a double-jacket reactor at 60 °C under mechanical stirring. First, the NMP was heated up to the desired temperature, and then PSU was slowly added to the reactor. Once PSU is completely dissolved, PVP was gradually added to the mixture to avoid the formation of lumps. The mixture was stirred mechanically overnight to ensure a good homogenization. Before membrane preparation, the polymer solution was sonicated for 2 h to remove any air bubbles trapped in the viscous solution.

In order to obtain a crosslinked PVP reference, pure PVP was treated with potassium persulfate (KPS) at 80 °C. First, 10 wt.% PVP was dissolved in water for 24 h under stirring at room temperature, then 20 wt.% of KPS was added to the solution and the mixture was purged for 30 min with nitrogen to remove the oxygen which can act as a persulfate radical scavenger. The mixture was heated at 80 °C and after 1 h, an orangey gel was formed and the reaction was stopped. The gel was dried in an oven at 60 °C for 24 h and further crushed in a mortar to obtain a powder.

#### 2.2.2. Membrane Preparation

The membranes were prepared via the non-solvent phase inversion method. First, the homogeneous polymer dope solution (PSU/PVP) was cast onto clean glass support and then submerged in a coagulation bath containing water as nonsolvent. Before casting, the glass supports, the knife, and the polymer dope solution were heated at 50 °C. The membranes were cast using a 250 µm thickness knife and a speed of 20 cm s^−1^. After casting, the membranes were introduced into a non-solvent bath (distilled water) at 40 °C for 30 min to induce the phase separation process. In this step, most of the solvent leaches from the membrane to the coagulation bath forming a porous membrane. Then, membranes were transferred into a water-washing bath at room temperature for 24 h to remove any remaining solvent. The main function of water washing is to allow the free PVP to diffuse out of the membrane in order to release the porosity. Membranes obtained at this point are used for FTIR measurements and are nominated as “reference”, since all of the membranes are fabricated the same way. The membranes will then undergo the crosslinking procedure with a residual PVP content similar to those of the reference membrane. This residual PVP is similar in all of the membranes, regardless of the crosslinking conditions they will follow afterwards.

#### 2.2.3. Crosslinking Process

After water washing, the membranes were cut into circles of about 2.27 cm^2^ of surface and soaked in KPS solution to carry out the crosslinking reaction. The solution was bubbled with nitrogen to remove oxygen that can act as a quencher of persulfate radicals (${\mathrm{SO}}_{4}^{{•}^{-}}$). These radicals are formed when the solution is heated over 40 °C and are responsible for PVP chain crosslinking. The temperature (50 and 80 °C), the reaction time (30 min, 1, 2, 4, and 8 h), and the concentration of crosslinking agent (0, 3, and 50 g/L) play a role in the crosslinking reaction. Thus, these parameters will be tuned to find the best crosslinking conditions. For each reaction time, both temperature and crosslinking agent concentration were screened. Table 1 summarizes all the selected conditions. After crosslinking treatment, the membranes were washed several times in water to remove any remaining persulfate traces. The conditions were chosen neatly based on patents concerning the cross-linking of PVP in PVDF membranes [20,21]. We will refer along the text to treated membranes to those membranes which have been in contact with cross-linking agent during the reaction, and thermally-treated membranes to those membranes which undergone the same thermal treatment but without cross-linking agent.

#### 2.2.4. Characterization Techniques

##### Structural and Chemical Characterizations

The morphology and chemical structure of the as-prepared PSU/PVP membranes were analyzed. Fourier transform infrared (FTIR) spectra were recorded with a NEXUS (Thermo Fisher Scientific, Waltham, MA, USA) instrument equipped with an attenuated total reflection (ATR) accessory in the frequency range of 600–4000 cm^−1^. The ATR-FTIR spectra were recorded with a resolution of 4 cm^−1^, and the average of the signals from 64 scans was taken. PVP mass percentage was estimated taking into account the ratio between the PSU and PVP peak areas. It is assumed that the PSU peak area is constant and does not change after the treatment. The morphology of both the membranes’ cross-sections and surfaces was observed using a Hitachi S4800 (Tokyo, Japan) scanning electron microscopy system (SEM). Before analysis, membranes were coated with platinum using an ion sputter coater. Water contact angles (WCA) were measured using a Digidrop instrument purchased from GBX Scientific LTD (Dublin, Ireland) and equipped with a B-CAM-21-BW (CCCIR) monochrome camera and a Led R60 lamp CONRAD. Approximately 3.0 μL of deionized water was deposited onto the membrane surface using a needle. The images were recorded and treated using Digidrop software (GBX Scientific LTD, Dublin, Ireland). The results are the average of 5 measurements. Membrane permeability was measured using a PRM-2000-LL-R porometer (IFTS, Foulayronnes, France) with a membrane-active surface area of 2.27 cm^2^ by solvent permeability. The pure water flux (*J_PW_*) was measured for each membrane by circulating pure water through the membrane system using an applied pressure range of 0−1 bar, each point was obtained by measuring the flow passing through the membrane during 60 s.

The *J_PW_* (L·h^−1^·m^−2^) was calculated using the following formula:

$$J_{\mathrm{PW}}=\frac{Q}{A}\;(L\cdot h^{-1}\cdot m^{-2})$$

where Q (L·h^−1^) is the amount of water that passed through the membrane and A (m^2^) is the membrane area. The permeability was determined from the slope of a linear fitting when the variation of *J_PW_* is represented versus the applied pressure. The membranes’ tensile strength was measured using a Zwick Roell (Ulm, Germany) (type 5 kN ProLine) with a force sensor XforceHP (Zwick Roell, Ulm, Germany)with a nominal capacity of 10 N. For the analysis, the membranes were soaked in a water bath and the excess water was removed before performing the analysis.

## 3. Results and Discussion

### 3.1. Investigation of PVP Cross-Linking

#### Attenuated Total Reflectance Fourier Transform Infrared Spectroscopy (ATR-FTIR)

**Figure 2:**
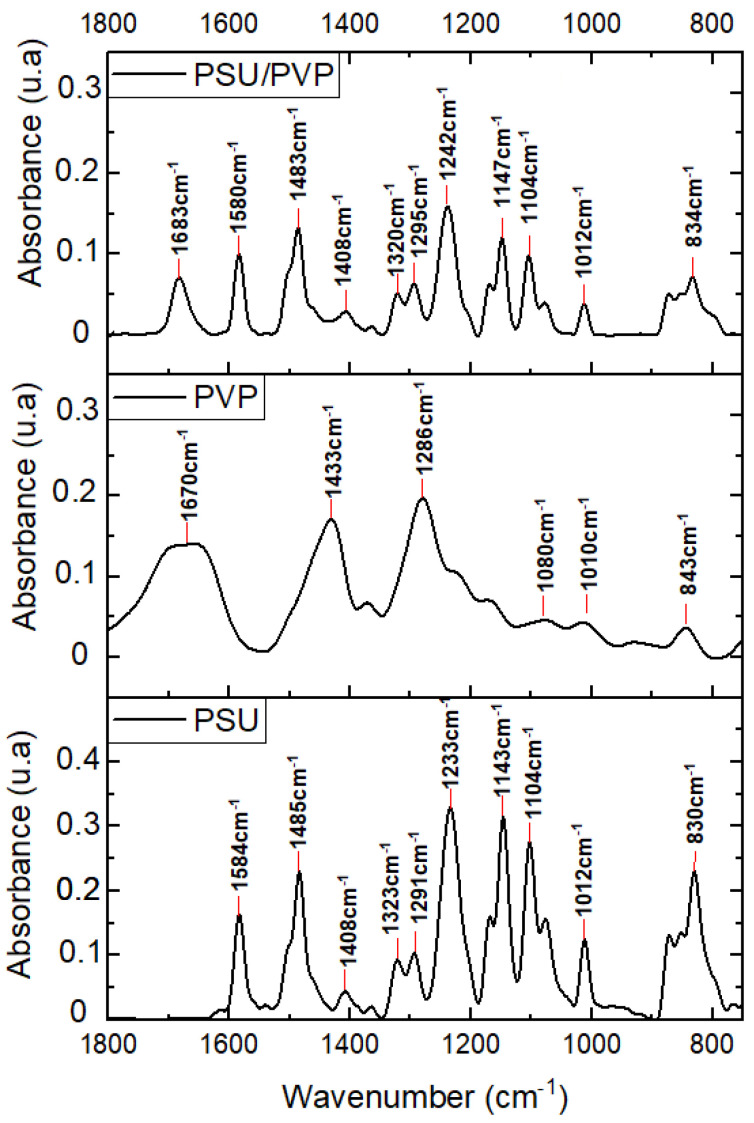
FTIR spectra of raw PSU and PVP polymers and PSU/PVP non-treated membrane.

The investigation of the intermolecular and intramolecular reactions between the functional groups of the polymers induced by the crosslinking reaction was performed by ATR-FTIR analysis. First, PVP powders and thermally-treated membrane were characterized to identify the main peaks. The main absorption bands of PVP are shown in Figure 2. PVP in the form of powder shows a characteristic peak at 1660 cm^−1^ corresponding to the amide I band of the pyrrolidone ring of PVP. Due to the hygroscopic nature of pure PVP, a shoulder can appear at 1646 cm^−1^, displaying water traces. An in-plane bending C-H band is observed at 1370 cm^−1^, and two vibrational modes can be detected, CH_2_ wagging and C-N stretching around 1290 cm^−1^. The vibrational mode of the C-C bond can be also observed with a band appearing at 1020 cm^−1^.

In this study, FTIR analyses will focus on the amide I band to evaluate the crosslinking effects of PVP on the PSU membranes. By comparing the PSU/PVP and PVP powder spectra, it appears that amide I band of the pyrrolidone ring of PVP is observed as well in PSU/PVP membrane at the same wavenumber (1660 cm^−1^), and another peak appears at 1580 cm^−1^ corresponding to PSU. In the case of pure PVP powders, the 1660 cm^−1^ peak is wider than in the spectrum of PVP in the membrane. This is due to stronger intramolecular interactions between PVP molecules in pure PVP; however, in the case of PVP in PSU these interactions are weaker.

**Figure 3:**
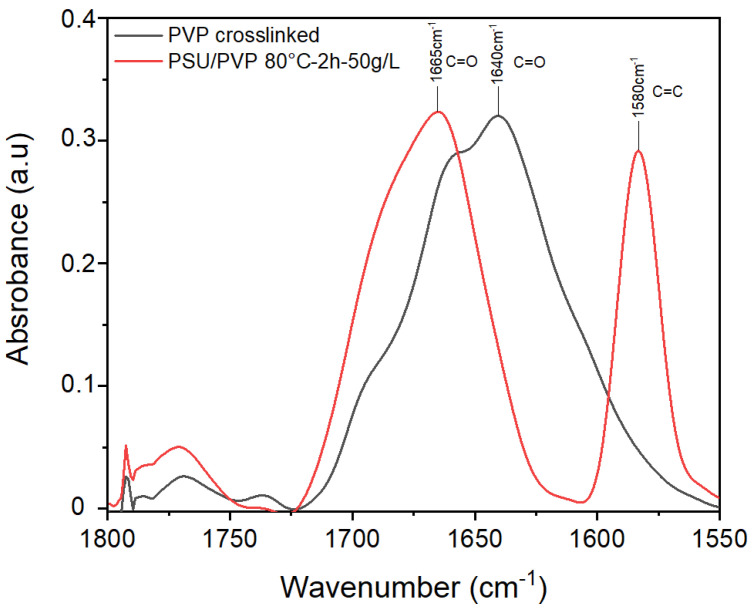
FTIR spectra of crosslinked PVP and PSU/PVP treated membrane.

In the case of pure PVP, the powder obtained after crosslinking with KPS was analyzed. The ATR-FTIR spectrum (Figure 3) shows one broad peak around 1660 cm^−1^ corresponding to the amide I band of the pyrrolidone ring. Different shoulders are observed in this peak after crosslinking treatment: a first shoulder appears at 1650 cm^−1^, a second shoulder at 1670 cm^−1^, and a third shoulder at 1700 cm^−1^. The downshift of the amide I band of the pyrrolidone ring from 1660 cm^−1^ to 1650 cm^−1^ is attributable to the formation of the hydrogen-bonded state of the C=O group in presence of water. The new band at 1700 cm^−1^ might be ascribed to C=O stretching modes of succinimide rings that are present in PVP after the crosslinking reaction as evidence of crosslinking [22]. Crosslinked PVP is compared to PSU/PVP membrane treated at 80 °C for 2 h in presence of 50 g/L of KPS. A similar peak shape is observed in the band corresponding to the amide I band of the pyrrolidone ring of PVP. In this case, the downshift of the amide I band of the pyrrolidone ring is not observable, only the shift to higher wavenumbers, indicating a change in the carbonyl environment and the apparition of new shoulders indicating the formation of succinimide groups after crosslinking reaction are visible.

**Figure 4:**
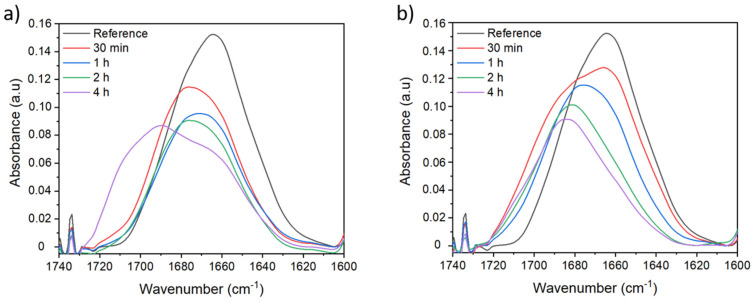
ATR-FTIR spectra of PSU/PVP non-treated membrane (reference) and membranes treated at 80 °C with (**a**) 3 g/L and (**b**) 50 g/L of KPS at different lengths of time.

In order to find the optimum conditions to carry out the crosslinking reactions at different durations, the initial KPS concentration and two different temperatures were evaluated. First, the reactions were carried out at a fixed temperature of 80 °C. In the non-treated membrane spectrum, two bands can be distinguished when the peak corresponding to the amide I group of the PVP is deconvoluted: the first one at around 1660 cm^−1^ and a second one at around 1680 cm^−1^. The band at 1660 cm^−1^ can be attributed to the amide I band of the pyrrolidone ring, the intensity of the peak decrease while increasing the reaction time from 30 min to 4 h (Figure 4a). This fact might be related to PVP leaching out from the membrane into the KPS solution by diffusion, thereby the amount of PVP on the membrane’s surface is reduced. Additionally, a new shoulder appears around 1700 cm^−1^ at a long reaction time. As mentioned above, this band might correspond to the formation of the succinimide group due to the crosslinking reaction. The three bands observed in the PVP peak (1660, 1680, and 1700 cm^−1^) can be attributed to three different forms of PVP. We have hypothesized that the first band at 1660 cm^−1^ could be attributed to the free PVP in the membrane and this PVP has no direct interaction with PSU. The second band at 1680 cm^−1^ could be attributed to the PVP linked to PSU. The new band appearing at 1700 cm^−1^ could be attributed to crosslinked PVP. We can also say that free PVP follows the crosslinking reaction since we observe that the shoulder at 1660 cm^−1^ decreases its intensity and the new shoulder appears at 1700 cm^−1^ after crosslinking.

In the case of membranes treated at 80 °C in presence of 50 g/L of crosslinking agent, a similar behavior to those treated at 3 g/L is observed (Figure 4b). The peak intensity decreases over the reaction time due to leaching effects; however, under these conditions, the band corresponding to amide I of the pyrrolidone ring presents new shoulders appearing at higher wavenumbers (around 1700 cm^−1^) which are already observable at a short reaction time. This new band might correspond to the formation of the succinimide group due to crosslinking reaction. Thus, at higher crosslinking agent concentration, the crosslinking effects are visible after 30 min reaction. It appears that at 2 h reaction time, the peak intensity is slightly reduced and a higher crosslinking effect is observed. A kind of competition may exist between the leaching out of PVP from the membrane and the crosslinking reaction.

**Figure 5:**
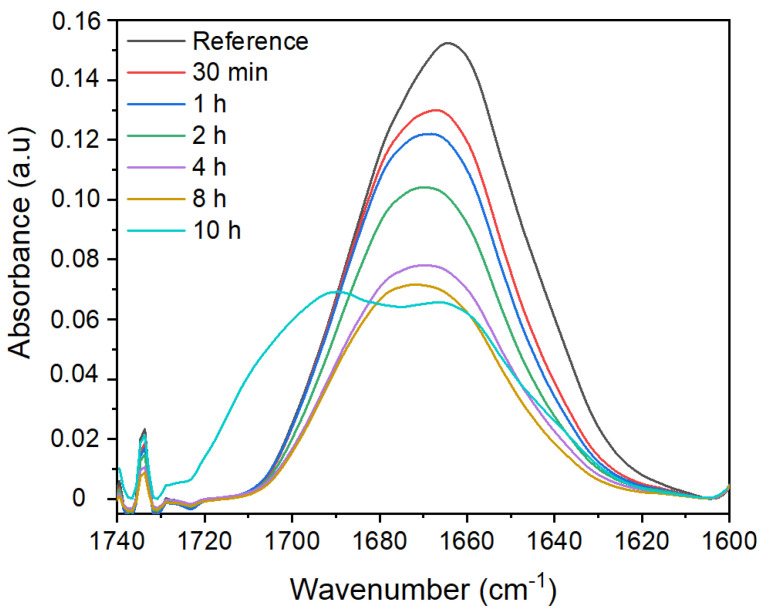
ATR-FTIR spectra of PSU/PVP non-treated membrane (reference) and membranes treated at 50 °C with 50 g/L of KPS at different lengths of time.

On other hand, the crosslinking reaction was carried out at 50 °C. This temperature was chosen as according to literature, radicals start to be formed above 40 °C and also contribute to energy saving. The FTIR spectra of membranes treated at 50 °C with 50 g/L are shown in Figure 5. Any sign of crosslinking is found for the peak corresponding to the amide I band of the pyrrolidone ring at a short time under the previously mentioned conditions. However, when the reaction is carried out for 10 h, we observe that the peak presents a shoulder at around 1700 cm^−1^, indicating that the environment of the carbonyl group has been modified probably due to the creation of succinimide groups resulting from crosslinking reaction. It is also observed that PVP peak intensity decreases over time due to leaching effects.

**Figure 6:**
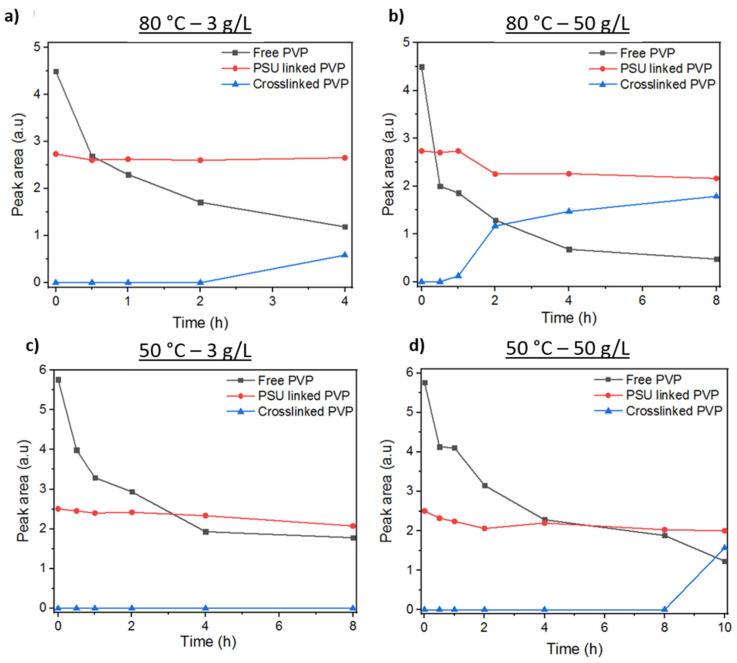
Evolution of PVP deconvoluted peaks of membranes treated at (**a**) 80 °C and 3 g/L of KPS, (**b**) 80 °C and 50 g/L of KPS, (**c**) 50 °C and 3 g/L of KPS, and (**d**) 50 °C and 50 g/L KPS.

In order to evaluate the peak area corresponding to the different bands of PVP, the main PVP peak has been deconvoluted into three different bands. We have hypothesized that these bands correspond to free PVP (band at 1660 cm^−1^), PSU-linked PVP (band at 1680 cm^−1^), and crosslinked PVP (band at 1700 cm^−1^). In the case of membranes treated at 80 °C and 3 g/L of KPS, Figure 6a displays the evolution of the areas of each peak as a function of the reaction time. The curve corresponding to PSU-linked PVP is rather constant over time. The free PVP curve decreases over time due to leaching. Furthermore, for a long reaction time (4 h), free PVP decreases at the same time as crosslinked PVP increases. We hypothesize that free PVP reacts preferentially with KPS. For the membranes treated at 80 °C and 50 g/L of KPS (Figure 6b), the evolution of the peak areas is similar to membranes treated with lower KPS concentrations. The peak area corresponding to the PVP strongly linked to the PSU is rather constant. However, the peak area corresponding to free PVP decreases more sharply during the first 30 min of the reaction while the peak area corresponding to crosslinked PVP starts to increase after this time. There was also a decrease in the concentration of all species during the first 30 min, probably due to their superficial leaching. Similar to membranes treated at 80 °C, for those treated at 50 °C, the peak attributed to PVP has been deconvoluted (Figure 6c,d) as well in three different bands corresponding to free PVP (band at 1660 cm^−1^), PSU-linked PVP (band at 1680 cm^−1^) and crosslinked PVP (band at 1700 cm^−1^). It is observed that at low concentration (3 g/L), the free PVP is reduced but no increase in crosslinked PVP is noticed. In the case of higher KPS concentration (50 g/L), the same trend is observed for free PVP but in this, case an increase in the crosslinked PVP is noticed at a long reaction time (10 h).

It is known from the literature that free sulfate radicals (${\mathrm{SO}}_{4}^{-\cdot }$) are formed by the thermal decomposition of persulfate salts in neutral or basic solutions above 40 °C according to the mechanism proposed by Bartlett [23] (Equations (1)–(3)). They also demonstrated that the persulfate decomposition reaction follows a first-order character at 79.8 °C and initial persulfate concentrations between 0.00240 and 0.0134 M. No evidence was found that radicals normally involved in its aqueous decomposition could induce the decomposition of persulfate.

(1) $$S_{2}O_{8}^{2-}\overset{k_{1}}{\to }2{\mathrm{SO}}_{4}^{-\cdot }$$

(2) $${\mathrm{SO}}_{4}^{-\cdot }+H_{2}O\overset{k_{2}}{\to }{\mathrm{HSO}}_{4}^{-}+\mathrm{OH}\cdot$$

(3) $$2\mathrm{OH}\cdot \overset{k_{3}}{\to }H_{2}O+\frac{1}{2}O_{2}$$

Indeed, the crosslinking effects can be observed for short time at 80 °C according to persulfate kinetics. The fact that any crosslinking effects are observed at 50 °C for a short time might be explained by Arrhenius’ law (Equation (4)). A widely used rule-of-thumb for the temperature dependence of a reaction rate is that a ten-degree rise in temperature approximately doubles the rate. Thus, if the temperature is reduced from 80 °C to 50 °C, it means that the persulfate takes more time to be decomposed into its radicals or they are still at a low concentration at that temperature. Therefore, after 10 h the persulfate ions had enough time to be formed and crosslinking effects are observed.

(4) k=Ae−EaRT

where k is the rate constant, E_a_ is the activation energy, R is the gas constant, A is a constant called the pre-exponential factor, and T is the temperature.

**Figure 7:**
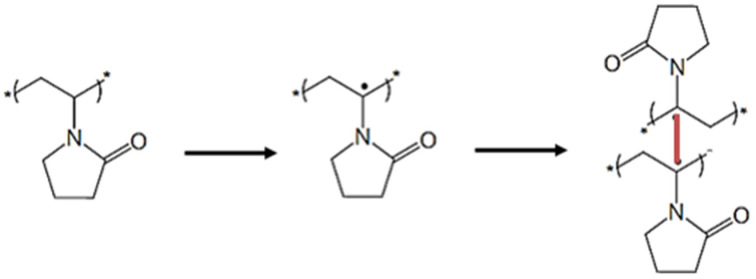
Schema of two PVP molecules crosslinking.

Crosslinking of PVP with persulfate involves the abstraction of a hydrogen atom from the polymer chain by an SO_4_^−^ or OH· radical. This hydrogen atom can be abstracted from three different sites; the most stable radical will be created when the hydrogen is abstracted from the carbonated chain [24]. The radical formed on the polymer chain (macroradical) can move by molecular or segmental diffusion to another macroradical and form a stable covalent crosslink (Figure 7). In addition to crosslinking, other reactions may occur, the macroradical may rearrange to a more stable state by chain scission via disproportionation in the neighborhood of the unpaired electron [16]. Other competing reactions may take place, such as polymerization of the residual monomer, deactivation of macroradicals by reaction with sulfate radicals, or deactivation due to the formation of water and oxygen from two hydroxyl radicals. The reaction is carried out under a nitrogen atmosphere, since the presence of oxygen may decay the macroradicals by oxidative degradation.

**Figure 8:**
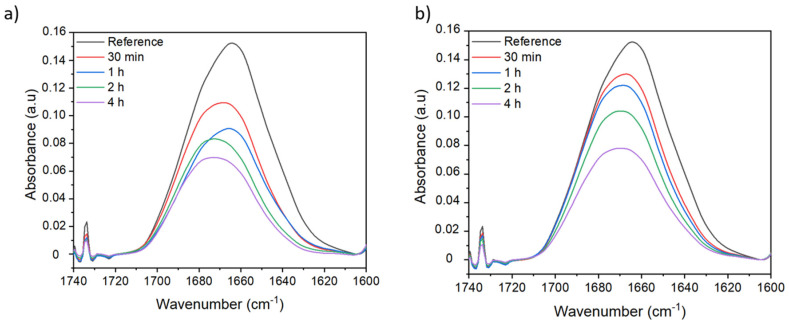
ATR-FTIR spectra of PSU/PVP non-treated membranes at (**a**) 80 °C and (**b**) 50 °C at different lengths of time.

In order to evaluate the kinetics of PVP leaching, the reaction was carried out under the same conditions, 50 and 80 °C at different lengths of time but without crosslinking agent. FTIR spectra show the typical peak at 1660 cm^−1^ corresponding to the carbonyl group of PVP (Figure 8). The peak intensity decreases over time indicating that leaching occurs during the treatment. In the case of membranes treated at 80 °C (Figure 8a), the leaching is more important than in membranes treated at 50 °C (Figure 8b).

**Figure 9:**
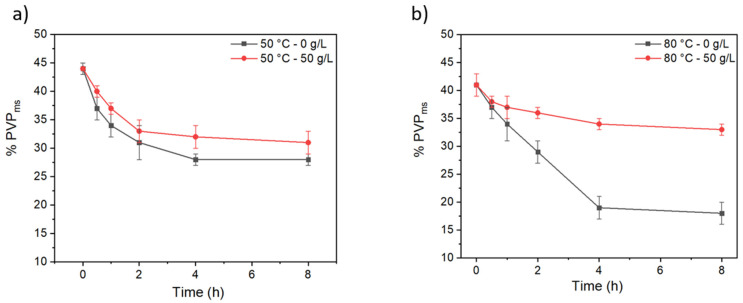
Residual PVP in membranes as a function of the crosslinking time of membranes treated at (**a**) 50 °C and (**b**) 80 °C thermally-treated and treated with 50 g/L of crosslinking agent.

PVP mass percentage (%PVP_ms_) was calculated from FTIR spectra by analyzing the ratio between the PVP and PSU peak areas, this last is supposed to be constant all over the treatment since it should not react with persulfate ions. The initial concentration of each polymer has been taken into account to calculate the final mass percentage of PVP after treatment. The %PVP_ms_ obtained from FTIR peak area analysis are represented in function of reaction time. Thermally-treated membranes at 50 °C and those treated with 50 g/L follow the same trends (Figure 9a): PVP mass percentage decreases linearly until reaches a plateau. In presence of KPS, crosslinking takes place at a long reaction time and it is observed that the amount of PVP barely increases when compared to the non-crosslinked membrane. Concerning the membranes treated at 80 °C, we observe two different trends (Figure 9b). On one hand, only those membranes thermally-treated follow zero-order kinetics with a linear dependence over time. However, membranes treated with 50 g/L present different kinetics after 30 min reaction. Persulfate radicals are formed under these conditions and can abstract hydrogens from PVP and start the crosslinking reaction. Thus, the amount of PVP in the membranes’ surface remains more important compare to the thermally treated membranes, indicating that crosslinking reaction is more important than PVP leaching out from the membrane.

### 3.2. Mechanical Properties

**Figure 10:**
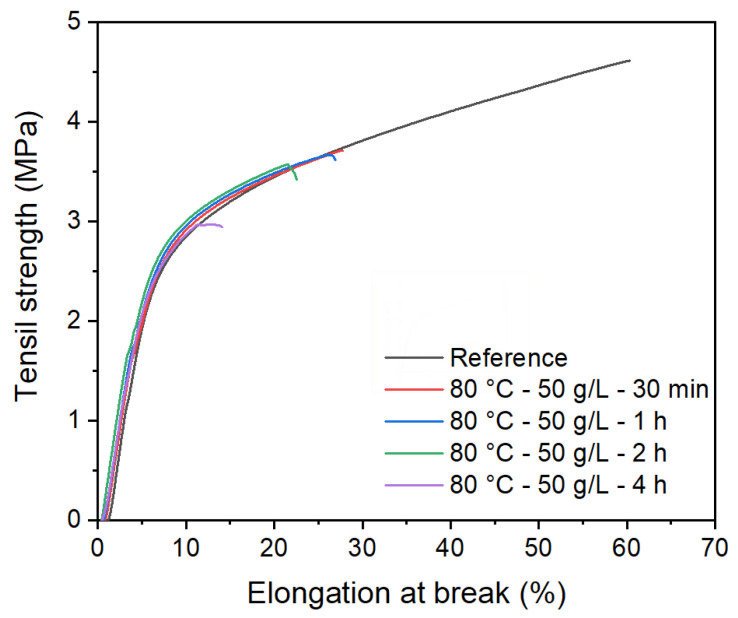
Stress–strain curves for non-treated membrane and membranes treated at 80 °C with 50 g/L at different lengths of time.

Stress–strain curves for non-treated membrane and two cross-linking treatments (80 °C, 50 g/L, 2 h and 80 °C, 50 g/L, 4 h) are presented in Figure 10, they display a viscoelastic behavior, with an elastic domain first followed by a plastic domain.

**Figure 11:**
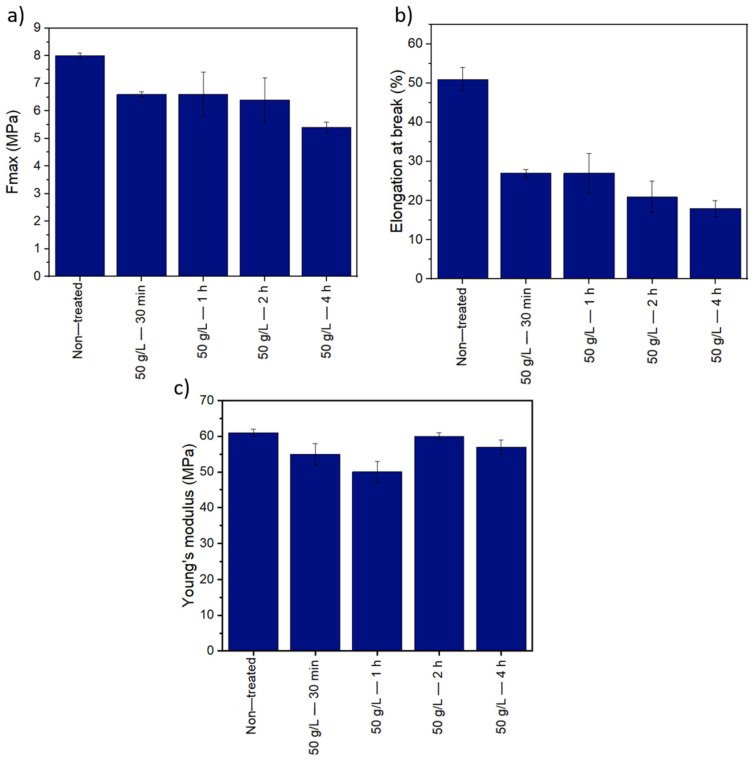
Mechanical properties of non-treated membranes and membranes treated at 80 °C with 50 g/L of KPS at different length of time: (**a**) tensile strength (**b**) elongation at break, and (**c**) Young’s modulus.

Young’s modulus, tensile strength, and elongation at break ratio values are represented in Figure 11. No significant differences are found in terms of Young’s modulus between the treated and non-treated membranes; thus, we can say that the elastic domain at low deformation of the membrane remains unchanged. This means that the PSU polymer phase is not significantly affected by the treatment applied to the membrane to induce the cross-linking reaction (80 °C and 50 g/L KPS). However, the plastic domain is reduced with increasing time treatment. Results show that the tensile strength and elongation at the break of treated membranes display lower values than treated membranes. These results indicate that crosslinked membranes tend to become more brittle compared to non-crosslinked membranes. This may be related to the PVP crosslinked content in the membrane, as evidenced by FTIR measurements. The cross-linking network might hinder the motion of molecules, limiting the sliding of polymer chains when the membranes are subjected to tensile load and reducing the percentage of elongation as a result [25]. At a longer reaction time, the crosslinking is more important; this is reflected in mechanical properties by the elongation at break: the membrane treated for 4 h is more brittle than the one treated for 2 h. Limiting the crosslinking reaction time allows maintaining the ductility of the membrane.

### 3.3. Physico-Chemical Characterizations

#### 3.3.1. Scanning Electron Microscopy (SEM)

**Figure 12:**
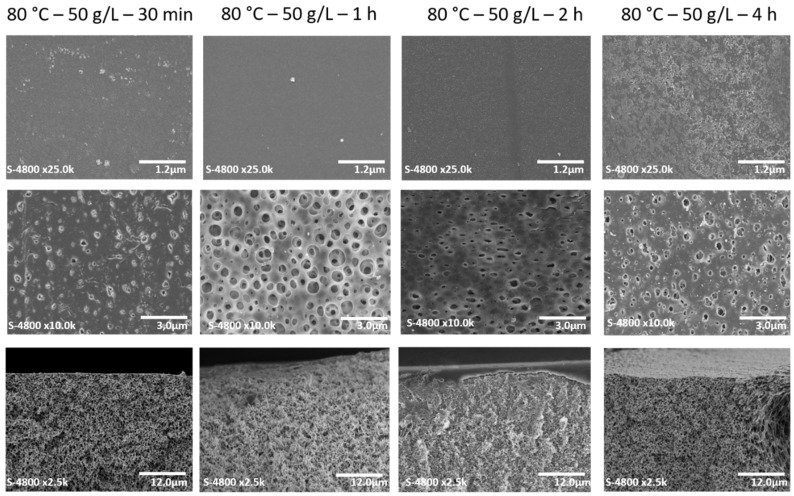
Top images correspond to top surfaces, middle images correspond to bottom surfaces and bottom images correspond to cross-sections of PSU/PVP membranes treated at 80 °C with 50 g/L of KPS at different reaction times.

SEM analyses were carried out on PSU/PVP membranes in order to determine their morphology. Cross-sections, top and bottom surfaces images of membranes treated at 80 °C with 50 g/L of KPS at different reaction times are shown in Figure 12, respectively. Similar morphologies are observed in membranes treated with KPS and those treated at the same temperature without KPS. Top surfaces do not present porosity at the applied SEM magnification. All membranes present porosity at their bottom surface. The pore size of both intern and extern surfaces was analyzed by image treatment using Fiji software.

In the case of the top surface for both reaction times (30 min and 4 h), we observe that the pore size slightly increases while increasing the crosslinking agent concentration (Table 2). The thermally-treated membrane does not present porosity at its top surface. The addition of KPS favors the opening of surface pores, increasing the reaction time. The KPS concentration increases the pore size; this might be attributed to the fact that since PVP acts as a porogen agent, it leaches out from the membrane and forms pores. The same trend is observed for the membranes treated at 80 °C for 4 h: when increasing the reaction time and KPS concentration, the pore size at the top surface increases. It is also observed that increasing the KPS concentration leads to a larger pore size distribution. The thickness of the selective layer was estimated using Image J software, using a larger set of SEM micrographs with higher magnification. The results show that the treatment with KPS reduces the thickness of the active layer (Table 2). This behavior is influenced by both temperature reaction and KPS concentration. At higher temperatures, the selective layer becomes thinner, and the same trend is observed when increasing the KPS concentration.

#### 3.3.2. Water Contact Angles Measurements

**Figure 13:**
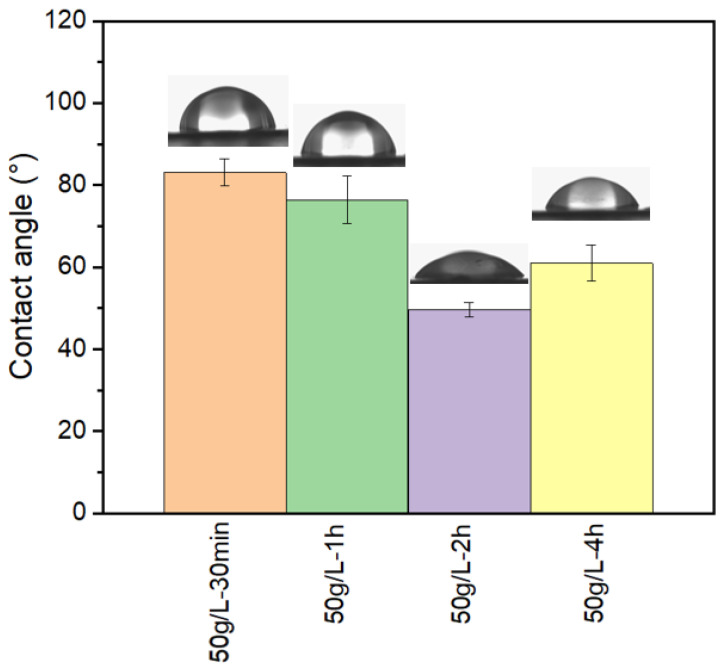
Contact angles measurements of crosslinked PSU/PVP membranes at 80 °C.

The main parameter that characterizes the hydrophilicity of membranes is the contact angle of water on the membrane material. However, sometimes it is hard to reliably measure reliably the contact angle directly on filtration membranes since they have complex surface morphology and significant porosity. The membranes’ hydrophilicity was evaluated through water contact angle measurements at room temperature. Figure 13 shows the contact angle values of PSU/PVP membranes treated at 80 °C with 50 g/L of KPS at various lengths of time. A trend is observed for the membranes treated with the crosslinking agent; when increasing the reaction time, the membrane’s contact angle decreases over time. This fact might be attributed to PVP leaching out from the membrane. At a short reaction time, the PVP can leach from the membrane and there are not enough persulfate ions to react with the PVP. When increasing the reaction time, there are more persulfate ions available to react with PVP, thus the PVP is crosslinked at the surface and hydrophilicity increases. However, after 4 h reaction, since the crosslinking reaction competes with the leaching, in this case leaching is more important than crosslinking, and thus the contact angle increases since we have less PVP at the membrane surface. After 4 h reaction, the amount of PVP at the surface has decreased, and thus the contact angle of the membrane has increased.

In order to understand the trend of the contact angle measurement, the amount of PVP in mass at the top surface has been estimated using ATR-FTIR spectra as well as the %PVP in mass at the bulk membrane using thermogravimetric analysis (TGA) (Table 3). The results of %PVP_ms_ in the membrane surface show that the amount of PVP available at the surface increases for a longer reaction time. This trend corresponds to the trend observed with contact angle measurements, at a longer reaction time the membrane surface becomes more hydrophilic. The membrane treated for 2 h presents the lower contact angle as well as the higher value of %PVP_ms_ at the surface. In the case of %PVP_ms_ obtained using TGA, the membrane treated for 2 h presents also the higher amount of PVP in the membrane bulk.

#### 3.3.3. Pure Water Permeability

The results of water permeability of the treated membranes are shown in Table 4. The membranes treated for 30 min with 50 g/L of KPS present a high permeability but with a higher standard deviation when compared to the membranes that were treated for longer times. Then, the water permeability of the membranes decreases with increasing reaction time. To understand the effect of the cross-linking treatment on the water permeability, it has been also measured with membranes treated at the same reaction time in the water at 80 °C but without KPS (Table 4). The results show that at any crosslinking reaction time, the permeability of the KPS treated membranes increases compared to those kept in water without KPS. We can thus conclude that crosslinking PVP at the membrane surface using KPS as crosslinking agent increases membranes’ water permeability.

There are two competitive phenomena, both useful for the membrane elaboration: the PVP leaching giving rise to the porosity and the PVP cross-linking that enhances the membrane surface hydrophily. FTIR analyses showed that PVP crosslinking was not achieved at the membrane surface within a duration of 30 min, whereas at the same time the treatment at 80 °C with KPS induces the PVP to leach out from the membrane. That may give a high permeability value but it does not guarantee that the membranes can stand on this good performance with time. The PVP leaching will make the membrane less hydrophilic under operation. The crosslinking reaction of PVP onto the membrane hinders the leaching of PVP out from the membranes and results in more stable membranes over time. Even if FTIR measurements depict traces of free PVP in crosslinked membranes, the total organic content analyses performed on the permeate after water filtration did not display any trace of organic compound.

## 4. Conclusions

PSU/PVP membranes were prepared by the non-solvent induced phase separation (NIPS) process. A thermal crosslinking reaction using KPS as a crosslinking agent was performed to immobilize PVP at the membrane’s surface to increase its hydrophilicity. ATR-FTIR and contact angle measurements confirmed the immobilization of the PVP on the PSU membrane. We have demonstrated that temperature has a significant influence on the crosslinking reaction, since persulfate radicals require a temperature of up to 40 °C to be formed. Furthermore, persulfate radicals display different kinetic rates depending on the temperature of the reaction. At low temperatures (50 °C), persulfate radicals take about 10 h to be formed and active to initiate the crosslinking reaction; however, at high temperatures (80 °C), this time is reduced to 1 h. A compromise between the leaching \ of PVP from the membranes increasing with reaction time and the PVP crosslinking reaction, strongly affected by temperature and KPS concentration, has to be established. Contact angle measurements indicate that at 80 °C and with 50 g/L KPS, the membranes display higher hydrophilic behavior than non-treated membranes for 2 h treatment. It also evident that PVP is crosslinked at the membrane’s surface. Moreover, the present study displays that the treatment at 80 °C and with 50 g/L KPS increases membrane water permeability. An inversion in the trend of the properties is observed when the crosslinking duration is 4 h, as a longer treatment will favor the extraction of PVP at the expense of surface hydrophilicity and of permeability. Mechanical properties analysis confirms that PVP crosslinking reduces membranes’ plastic domain with a breakpoint for deformation higher than 20% and 10% for 2 h and 4 h treatment, respectively. All of these data, when combined with energy consumption, tend to conclude that the treatment at 80 °C for 2 h in presence of 50 g/L. KPS presents a good compromise: it increases the hydrophilicity and water permeability of the membrane surface and maintains good mechanical performances for deformation lower than 20%.

## Figures and Tables

**Table 1:** Crosslinking conditions of the PSU membranes.

Polymer	Time (h)	Temperature (°C)	[KPS] (g/L)
PSU	0.51 2 4 8 10 *	50	0 **
			3
			50
		80	0 **
			3
			50

* 10 h reaction has been performed only for the lower temperature condition (50 °C). ** We will refer to these membranes as thermally-treated membranes.

**Table 2:** Membranes’ pore size at the top and selective layer thickness (n.d. not determined).

Membrane	Top Surface Pore Size (nm)	Selective Layer Thickness (µm)
Non-treated	n.d	3.07 ± 0.85
80 °C, 0 g/L, 30 min	26 ± 4	0.36 ± 0.04
80 °C, 50 g/L, 1 h	25 ± 1	0.32 ± 0.06
80 °C, 50 g/L, 2 h	24 ± 0.1	0.26 ± 0.04
80 °C, 50 g/L, 4 h	24 ± 7	0.22 ± 0.02

**Table 3:** PVP mass percentage on the membrane surface and at the bulk membrane.

Membrane	%PVP_ms_ at the Surface	Total %PVP_ms_
80 °C, 50 g/L, 30 min	32.2 ± 1.6	7.6 ± 0.6
80 °C, 50 g/L, 1 h	25.9 ± 2.6	7.5 ± 0.4
80 °C, 50 g/L, 2 h	47.8 ± 0.6	9.6 ± 0.8
80 °C, 50 g/L, 4 h	44.3 ± 0.6	7.3 ± 0.4

**Table 4:** Water permeability of membranes treated at 80 °C with and without 50 g/L of KPS.

Membrane	P_0_ (0 g/L) (L·bar^−1^ h^−1^ m^−2^)	P (50 g/L) (L·bar^−1^ h^−1^ m^−2^)	P/P_0_
80 °C, 30 min	86 ± 24	539 ± 129	6.3
80 °C, 1 h	58 ± 4	330 ± 40	5.7
80 °C, 2 h	126 ± 32	268 ± 60	2.1
80 °C, 4 h	71 ± 8	195 ± 41	2.7

## Data Availability

All data is presented in the article.
